# Cytomegalovirus-specific CD8 T cells kill B16 melanoma cells in vivo when activated by bifunctional major histocompatibility class I - antibody fusion molecules (pMHCI-IgGs).

**DOI:** 10.1186/2051-1426-3-S2-P237

**Published:** 2015-11-04

**Authors:** Michael Munks, Victor Levitsky, Ann Hill, Hendrik Knoetgen

**Affiliations:** 1 Oregon Health & Science University, Portland, OR, USA; 2 Roche Pharmaceutical Research and Early Development, Schlieren, Switzerland; 3 Roche Innovation Center Penzberg, Penzberg, Germany

## 

Bispecific antibodies (BiAb), some of which simultaneously bind to tumor-associated antigens on tumors and activate the CD3 receptor of T cells, have shown promising results in both pre-clinical models and clinical trials. However, widespread activation of T cells can result in toxicity, while sometimes still failing to control or eradicate tumors. A related but alternative approach is to recruit virus-specific CD8 T cells to kill tumor cells. Cytomegalovirus is a species-specific herpesvirus that establishes lifelong, latent infection, with periodic episodes of reactivation. It is an appealing virus for this approach because both human CMV (HCMV) in humans and murine CMV (MCMV) in mice elicit very large numbers of virus-specific CD8 T cells, with a highly activated and differentiated phenotype, that persist for the life of the animal. Therefore we hoped to redirect these T cells to kill tumor cells. We previously engineered single-chain bifunctional fusion molecules that contained HCMV peptide-major histocompatibility class I (pMHCI) linked to a tumor-specific monoclonal antibody (pMHCI-IgG)[1]. These pMHCI-IgGs were able to able to sensitize tumor cells to killing by either cytotoxic T cell lines (CTL) or by CD8 T cells directly ex vivo.

To extend this work into a relevant small animal model, we engineered new pMHCI-IgGs that contained immunodominant MCMV epitopes linked to murine MHC. When the mAb domain was specific for the B cell marker CD20, these pMHCI-IgGs could activate MCMV-specific CD8 T cells in vitro, and administration to mice led to depletion of circulating B cells. After obtaining these proof-of-principle data, we next tested the efficacy of these bifunctional proteins against B16 melanoma cells, an aggressive, immunosuppressive, transplantable tumor. The IgG portion of the chimeric proteins was redesigned to confer specificity to B16 cells. When administered prophylactically, the pMHC-IgGs prevented B16 tumor formation. Importantly, similar protection was seen in the more stringent therapeutic setting, when B16 cells were allowed to establish themselves for three days before beginning treatment. Furthermore, hematological analyses indicated that there was no overt toxicity. In ongoing work, we are testing whether these bifunctional proteins cause CD8 T cell proliferation, which has the potential to amplify their effectiveness, and are testing the limits of these drugs' therapeutic efficacy.
